# Scaling up calcification, respiration, and photosynthesis rates of six prominent coral taxa

**DOI:** 10.1002/ece3.8613

**Published:** 2022-03-18

**Authors:** Jeremy Carlot, Héloïse Rouzé, Diego R. Barneche, Alexandre Mercière, Benoit Espiau, Ulisse Cardini, Simon J. Brandl, Jordan M. Casey, Gonzalo Pérez‐Rosales, Mehdi Adjeroud, Laetitia Hédouin, Valeriano Parravicini

**Affiliations:** ^1^ PSL Université Paris USR 3278 CRIOBE ‐ EPHE‐UPVD‐CNRS Perpignan France; ^2^ Laboratoire d’Excellence “CORAIL” Paris France; ^3^ CESAB ‐ FRB Montpellier France; ^4^ 8002 Australian Institute of Marine Science Crawley Western Australia Australia; ^5^ Oceans Institute The University of Western Australia Crawley Western Australia Australia; ^6^ PSL Université ‐ EPHE‐UPVD‐CNRS USR 3278 CRIOBE Papetoai French Polynesia; ^7^ Integrative Marine Ecology Department Stazione Zoologica Anton Dohrn National Institute of Marine Biology, Ecology and Biotechnology Napoli Italy; ^8^ Marine Research Institute University of Klaipeda Klaipeda Lithuania; ^9^ Department of Marine Science The University of Texas at Austin Marine Science Institute Port Aransas Texas USA; ^10^ ENTROPIE, IRD Université de la Réunion, Université de la Nouvelle‐Calédonie CNRS, Ifremer Perpignan France

**Keywords:** calcification, coral physiology, coral reefs, demographic dynamics, photosynthesis, respiration

## Abstract

Coral reefs provide a range of important services to humanity, which are underpinned by community‐level ecological processes such as coral calcification. Estimating these processes relies on our knowledge of individual physiological rates and species‐specific abundances in the field. For colonial animals such as reef‐building corals, abundance is frequently expressed as the relative surface cover of coral colonies, a metric that does not account for demographic parameters such as coral size. This may be problematic because many physiological rates are directly related to organism size, and failure to account for linear scaling patterns may skew estimates of ecosystem functioning. In the present study, we characterize the scaling of three physiological rates — calcification, respiration, and photosynthesis — considering the colony size for six prominent, reef‐building coral taxa in Mo'orea, French Polynesia. After a seven‐day acclimation period in the laboratory, we quantified coral physiological rates for three hours during daylight (i.e., calcification and gross photosynthesis) and one hour during night light conditions (i.e., dark respiration). Our results indicate that area‐specific calcification rates are higher for smaller colonies across all taxa. However, photosynthesis and respiration rates remain constant over the colony‐size gradient. Furthermore, we revealed a correlation between the demographic dynamics of coral genera and the ratio between net primary production and calcification rates. Therefore, intraspecific scaling of reef‐building coral physiology not only improves our understanding of community‐level coral reef functioning but it may also explain species‐specific responses to disturbances.

## Graphical abstract

Our paper provides information concerning 3 metabolic rates (i.e., photosynthesis, respiration, and respiration) of 6 prominent coral reef taxas from Mo'orea. Moreover, we show that defining these 3 functions might explain coral reef dynamics after perturbations.

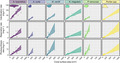

## 1 INTRODUCTION

Coral reefs are among the most diverse marine ecosystems and provide essential services to more than 500 million people worldwide (Hoegh‐Guldberg, 2011). While there is broad agreement on which processes are fundamental for reef systems, our capacity to quantitatively define a functional reef is still limited (Brandl, Rasher, et al., 2019; Hughes et al., 2017; Kennedy et al., 2013). Indeed, coral reef functioning is based on physiological processes at the organismal level that determine community‐level fluxes (Brandl, Rasher, et al., 2019). For example, scleractinian corals produce both carbon dioxide (CO_2_) and dioxygen (O_2_) through respiration and their symbiotic association with photosynthetic microalgae from the Symbiodiniaceae family (LaJeunesse et al., 2018). The coral host receives photosynthetically fixed carbon that may support up to 95% of its metabolism (Muscatine, 1990), including skeletal growth through calcification rates (Barnes, 1987; Barnes & Hughes, 1999; Birkeland, 1997; Muscatine, 1990). These basic physiological processes determine community‐level, elemental fluxes, and key functions, such as reef accretion (Howard et al., 2017). Therefore, accurate quantifications of species‐specific rates of calcification, respiration, and photosynthesis rate are necessary to estimate system‐wide functioning of coral communities (Madin et al., 2016).

In order to integrate empirically measured rates into assessments of reef functioning, one may use two main approaches. First, one may directly measure elemental fluxes at a community level through *in situ* incubations (e.g., Nakamura & Nakamori, 2009). This approach is the most accurate method to quantify fluxes, but it requires a huge effort in the field and cannot be applied over large spatial scales. The second approach is based on scaling individual‐level physiological processes at the community level. This approach benefits from an extensive literature on metabolic scaling, which examines the relationship between body size, metabolic rate, and ecological processes at different levels of ecological organization (Chave, 2013; Levin, 1992). While the metabolic rate of most taxa scales allometrically with body size (Brown et al., 2004), scaling for colonial animals, such as corals, remains unclear (Barneche et al., 2017; Hartikainen et al., 2014). The scaling of individual‐level physiological processes to the community level has been used to estimate large‐scale biomass production and nutrient cycling in coral reef fishes (Allgeier et al., 2014; Brandl, Tornabene, et al., 2019; Morais et al., 2020; Schiettekatte et al., 2020) as well as carbonate production and vertical reef accretion in coral assemblages (Page et al., 2017; Perry et al., 2012). A clear advantage of this approach over the direct assessment of elemental fluxes is that it can leverage widely available datasets on coral abundances or community structure. However, reliable estimates will inevitably depend on the availability and accuracy of physiological measurements conducted at the individual level across different sizes (Edmunds & Riegl, 2020).

Currently, it is not clear whether physiological rates scale allometrically (i.e., exhibiting varying rates across colony sizes) or isometrically (i.e., exhibiting constant rates across colony sizes) (Dornelas et al., 2017; Edmunds & Burgess, 2016; Jokiel & Morrissey, 1986; Vollmer & Edmunds, 2000). For example, recent researches demonstrate that the growth rate of large colonies is substantially lower than that of smaller coral colonies (Carlot et al., 2021; Edmunds & Burgess, 2016). However, it is still not clear whether allometric growth emerges from allometric scaling of calcification rates or partial mortality. In favor of allometric scaling, one hypothesis is that larger colonies may invest substantial energy in reproduction, which reduces the energy available for growth (Richmond, 1987). In favor of isometric scaling, larger colonies can experience higher partial mortality (e.g., localized tissue necrosis, overgrowth by other organisms and predation from parrotfishes), which may reduce apparent growth rates (Madin et al., 2020; Pratchett et al., 2015). The uncertainty surrounding allometric or isometric scaling in corals also applies to other physiological processes such as respiration and photosynthesis (Edmunds & Burgess, 2016). Therefore, understanding whether and why physiological rates scale isometrically or allometrically with colony size has important implications for our capacity to estimate community‐level processes and make recommendations regarding ecosystem functioning (Edmunds & Riegl, 2020).

In the present study, we quantify three primary physiological rates (i.e., calcification, respiration, and photosynthesis) for six coral taxa along a gradient of colony size to examine whether each species exhibits an isometric or allometric physiological pattern. Then, we scale our physiological rate estimates to the community level to estimate overall reef functioning.

## 2 MATERIALS AND METHODS

### 2.1 Coral species selection, preparation, and acclimation

From September 2018 to December 2018, we collected 384 coral colonies from six coral taxa: *Acropora hyacinthus* (*n* = 72), *Astrea curta* (*n* = 60), *Montipora verrilli* (*n* = 48), *Napopora irregularis* (*n* = 48), *Pocillopora* cf. *verrucosa* (*n* = 84), and massive *Porites* spp. (*n* = 72). These taxa are among the most abundant reef‐building coral species in Mo'orea, French Polynesia (Bosserelle et al., 2014). They also represent a large range of morphologies, such as tabular (*A*. *hyacinthus*), branched‐corymbose (*N*. *irregularis* and *P*. *verrucosa*), encrusting (*M*. *verrilli*), and massive (*A*. *curta* and *Porites* spp.). We were unable to distinguish massive *Porites* beyond the genus level because *P*. *lutea* and *P*. *lobata* are indistinguishable *in situ*. We sampled all coral colonies at a depth of 11–13 m on the reef slope of the northern coast of Mo'orea. Each week, we collected 60 corals colonies from 2 coral species. Before each coral collection, we recorded mean ambient seawater temperature and salinity *in situ* with temperature and salinity probes from Pyroscience (Pyroscience GmBH, Aachen, Germany), and at 12m depth, we measured the photosynthetically active radiation (PAR: 400–700 nm) with an underwater quantum sensor from LI‐COR Biosciences (LI‐COR Biosciences GmbH, Bad Homburg, Germany) three times per week at 2 pm. We collected colonies from the substratum using a hammer and chisel and transported them to the lab in a cooler filled with unfiltered seawater. Transportation took approximately 15 min.

### 2.2 Tank preparation

**FIGURE 1:**
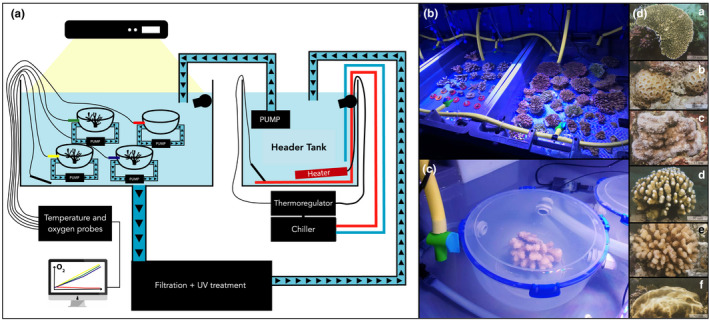
(a) Experimental set up of tanks. (b) Coral colonies in two tanks conditioned to reflect *in situ* environmental parameters. In the left tank, the coral colonies are *A*. *hyacinthus*, and in the right tank, they are *N*. *irregularis*. (c) *P*. *verrucosa* in an incubation chamber used to define calcification and gross photosynthesis rates. (d) Photos of the 6 coral species: a. *A*. *hyacinthus*; b. *A*. *curta*; c. *M*. *verrilli*; d. *N*. *irregularis*; e. *P*. *verrucosa*, and f. *Porites* spp. (from Bosserelle et al. (2014))

In the laboratory, we removed carefully epibionts or epiphytes. We visually assigned each colony to a size class: (S1) <100 cm^2^, (S2) 100–400 cm^2^, and (S3) >400 cm^2^ for further physiological measurements. Each week, we placed the 60 coral colonies (30 coral colonies of each species) into 2 to 4 recirculating tanks (with the dimensions 80 cm x 45 cm x 20 cm; Figure 1), which had the same environmental conditions (i.e., temperature, salinity, Ph, and light) as field conditions during sample collection. To evaluate any potential effect of stress on the colony during sampling, we collected thirty corals from the same species that we kept in the same tank (*n* = 10 for each size class), but only 12 colonies per species were used in the experiment. Following Edmunds and Burgess (2017), we gave the colonies 7 days to recover and acclimate and assumed that the acclimation was successful due to the low incidence of bleaching (only 2 coral colonies). At the end of the acclimation period, we incubated 12 coral colonies while placing a new set of 30 coral colonies in the acclimation tank (Figure 1). We ensured that each acclimatation tank had a different species from one week to the next to avoid tank effects. Every 3 days, the header tanks were re‐filled with water from the forereef and water was pumped into a buffer tank. Temperature and pH data were obtained every 2 s with probes from Neptune Systems APEX (Neptune Systems, Morgan Hill, USA) and Pyroscience (Pyroscience GmBH, Aachen, Germany). The probes were calibrated each week. To maintain constant conditions (i.e., pH between 8.1 to 8.3 and temperature between 25.5 to 30.2°C), we installed a chiller and heater in the buffer tank, and the water coming from the header tank was filtered and UV treated. Light intensity was regulated by artificial lights above all tanks, simulating high light‐intensity conditions 12m depth without any clouds (i.e., 350 μmol quanta m^−2^ s^−1^; Figure 1) for 12 h per day.

### 2.3 Respiration and photosynthesis

We assessed coral respiration and photosynthesis using continuous‐flow respirometry, where colonies were immersed in chambers connected to both a closed recirculating pump system and an open flush‐pump system to periodically record oxygen concentrations in the unfiltered seawater. Corals from size classes S1, S2, and S3 were incubated in 0.5 L, 1 L, and 4 L chambers, respectively, to maintain a similar ratio between incubation volume and colony size. Pumps were set at flow rates of 0.6, 2, and 7.5 L min^−1^, respectively, to maintain a low turbulent flow speed for each incubation chamber (i.e., 0.5 cm s^−1^; Edmunds & Burgess, 2017). For each set of respirometry measurements, we assessed four controls (empty chambers) and four corals of each size class (*n* = 12 colonies for each set of measurements) in both artificial light and dark conditions. For each set of measurements, we exposed colonies to light for three hours (i.e., 350 μmol quanta m^−2^ s^−1^), then we turned off the light and recorded O_2_ consumption 30 min later. We limited the dark phase to 1 h to prevent O_2_ concentrations from falling below 80% saturation (Kolb, 2018). O_2_ concentration was recorded with PyroScience FireSting optical oxygen meters (Pyroscience GmBH), which were factory calibrated. We removed the first thirty minutes of each set of measurements, which corresponded to the stabilization of the O_2_ concentration slopes in the closed stage of the system, and we included a chamber that was not populated with a coral colony to account for background bacterial respiration. Using these controls, we corrected O_2_ concentrations for each set of measurements, ultimately yielding two consumption profiles: one that corresponded to physiological activity in daylight (i.e., gross photosynthesis) and the other in nocturnal conditions (i.e., respiration). All oxygen fluxes are described in mg (O_2_) h^−1^. The respirometry system was soaked in sodium hypochlorite for 30 min after each set of measurements to minimize background respiration by the accumulation of microorganisms.

### 2.4 Calcification

We collected 50 ml of water from each incubation chamber and the control chambers at the beginning and end of the experiment, both in light and dark conditions. We stored the samples in sealed, opaque vials in the dark at 4°C for a maximum of 3 days. Then, we allowed them to stabilize for 2 h at room temperature (25°C) before processing. We carried out three titrations per sample to define total alkalinity using a Titrando 888 (Metrohm) and Titripur c(HCl) (with a concentration of 100 mmol L^−1^). We defined titration controls with water samples collected before coral incubations. We calculated calcification rates based on the difference between total alkalinity at the beginning and end of each incubation period (∆AT) (Dickson et al., 2007). Specifically, we assumed that one mole of CaCO_3_ is produced when alkalinity (∆AT) drops by two moles across a fixed time period (∆*t*) (i.e., −∆AT/2∆*t*), and then we multiplied the result with seawater density (ρ_sw_; i.e., 1.025 kg L^−1^). To obtain a calcification rate per surface area, we divided our result by coral surface area (for surface area calculations, see Section 2.5 Colony‐size estimation using photogrammetry). Finally, we converted the resulting value from mol cm^−2^ h^−1^ to g cm^−2^ h^−1^ based on the molar mass of CaCO_3_ (g mol^−1^).

### 2.5 Colony‐size estimation using photogrammetry

After each set of incubations, we took 100 to 200 overlapping high‐resolution photos (300 dpi) of each colony. The photos were used to construct 3D models using the Agisoft PhotoScan software (Agisoft, 2016), which allowed us to quantify the 3D living surface area of each colony (Harwin et al., 2015). We worked with 3D surface area rather than planar area to avoid overestimating coral calcification. To ensure reproducibility, we also defined the Coral Shadow Area (Grottoli et al., 2021) to expand the application of our estimates. All coral colonies (*n* = 384) were then placed in a large holding aquarium (for a maximum of 2 weeks) and ultimately returned to the outer reef.

### 2.6 Modeling physiological rates

Before analyzing the data, we removed data points if (a) a coral colony exhibited a negative calcification rate (i.e., dissolution), (b) the tank temperature dropped below 27°C or above 31°C (i.e., failure of the tank cooling or heating systems), or (c) the linear fit of O_2_ concentrations over time to quantify respiration or net photosynthesis rates exhibited an *R* ^2^ value lower than 0.8 (Kolb, 2018). Therefore, due to an equipment malfunction involving water supply in September, temperatures superseded 31°C at several time points. Consequently, for data analysis, we discarded measurements over those 4 weeks in September (i.e., 25% of the data, including 96 coral colonies). We removed an additional 8% of our data following the recommendation of Kolb (2018) and a further 2% of our data due to negative calcification rates. Following this quality control procedure, we retained 250 out of 384 (65%) of data points for the analysis.

We applied Bayesian models to estimate the relationship between colony surface area and each physiological rate on the natural log scale using the R package *brms* (Bürkner, 2017). Our models were specified with the following structure:

$$\ln\left(R_{S,i}\right)∼N\left(\mu_{S,i},\sigma \right)$$

$$\mu_{S,i}=\left(\ln\left(\alpha \right)+\zeta_{\left[S_{i},1\right]}\right)+\left(\beta +\zeta_{\left[S_{i},2\right]}\right)\ln\left(x_{i}\right)$$

$$\zeta =\left(\Omega Z\right)\delta_{s}$$

$$\mathrm{diag}\;\left(Z\right)=\;\sigma_{\zeta }$$

$$\beta \;∼\;N\left(0,\;5\right);\ln\left(\alpha \right)∼\;N\left(0,\;5\right);\;\sigma \;∼\;\Gamma \left(2,\;0.1\right);\;\delta_{s}\;∼\;N\left(0,\;1\right);\;\Omega \;∼\;\mathrm{LKJ}\left(1\right);\;\sigma_{\zeta }\;∼\;\Gamma \left(2,\;0.1\right)$$

where $\ln\left(R_{S,i}\right)$ is the natural logarithm of the rate of calcification (kg h^−1^), O_2_ consumption (mg h^−1^), or O_2_ production (mg h^−1^) of species S and individual i; $\ln\left(x_{i}\right)$ is the natural logarithm of live coral surface area (cm^2^); ln(α) is the among‐species average intercept on the natural log scale; β is the among‐species average size scaling slope (i.e., exponent on the natural scale); $S_{i}$ is a vector comprising s levels of species (*n* = 6), which, in turn, create a hierarchical matrix ζ of s rows and two columns, respectively, representing species‐level additive deviations from ln(α) and β; Ω is the Cholesky factor of the correlation matrix between the hierarchical effects, Z is the two‐by‐two diagonal matrix, for which the diagonal is a vector of among‐species standard deviations ($\sigma_{\zeta }$), and $\delta_{s}$ is an s‐by‐two matrix of standardized hierarchical effects. The prior sampling distributions were specified to follow Gaussian (N(location, scale)), Gamma (Γ(shape, inverse scale)), and log‐LKJ (LKJ(shape)). We ran our models with three chains, 5,000 draws per chain, and a warm‐up period of 2500 steps, thus retaining 7500 draws to construct posterior distributions. We verified chain convergence with trace plots and confirmed that *R*_hat_ (the potential scale‐reduction factor) was lower than 1.05 (Gelman et al., 1992). We obtained *R* ^2^ values of 0.92, 0.77, and 0.77 for the calcification rate model, respiratory rate model, and photosynthetic rate model, respectively (Table 1, Figure S1). We then divided our raw data by the respective surface area of each colony and plotted area‐specific rates. To calculate the posterior distribution of the scaling exponent of area‐specific rates against colony area, we used 1‐*β* (Figure S2).

**TABLE 1:** Point estimates and 95% credible intervals for fitted parameters based on Bayesian linear models estimating calcification, respiration, and photosynthesis rates based on colony size and species identity

	Calcification			Respiration			Photosynthesis		
Parameters	Mean	2.5%	97.5%	Mean	2.5%	97.5%	Mean	2.5%	97.5%
Fixed effects									
ln(*α*)	−6.126	−6.719	−5.486	−4.154	−5.565	−2.741	−3.971	−5.074	−2.907
*β*	0.881	0.792	0.966	1.074	0.796	1.351	1.033	0.800	1.256
Random effects									
SD of ln(*α*)	0.613	0.228	1.408	1.437	0.624	3.006	1.081	0.383	2.376
SD of *β*	0.075	0.006	0.199	0.281	0.100	0.638	0.221	0.050	0.519
Correlation of ln(*α*) and *β*	−0.58	−0.98	0.527	−0.602	−0.959	0.236	−0.507	−0.953	0.536

### 2.7 Community‐level scaling

To infer community‐level processes such as respiration, photosynthesis, and calcification rates, we used models that relate physiological rates to body size. Specifically, we tested whether the community‐level ratio between net photosynthesis and calcification rates changes according to variations in coral cover across a disturbance‐recovery cycle. This was completed under the hypothesis that the ratio between net photosynthesis and calcification may be a proxy for energy availability for functions other than growth (e.g., reproduction) (Rinkevich, 1989). We hypothesized that species with more residual energy after growth might be favored under disturbance.

To create these models, we combined two data sets: (a) a coral cover time series data set and (b) a coral colony size distribution data set from Mo'orea. The first data set was collected by the “*Service d’Observation CORAIL*” (http://observatoire.criobe.pf) and reports changes in coral cover in Mo'orea from 2004 to 2017. These data recorded coral cover variation at the genus level across a disturbance and recovery cycle. Indeed, Mo'orea experienced an *Acanthaster* cf. *solaris* outbreak from 2006 to 2009, followed by a cyclone in 2010, reducing live coral cover from approximately 50% in 2005 to 3% in 2010 (Carlot et al., 2020; Kayal et al., 2012). Following these disturbances, coral cover recovered to predisturbance levels by 2016 (Kayal et al., 2018). The second data set reports the size distributions of *Acropora*, *Pocillopora*, and *Porites* in Mo'orea (Kayal et al., 2018). The authors detected an almost identical colony‐size distribution among the three genera, so we assumed that *Montipora*, *Napopora*, and *Astrea* followed the same size distribution.

For each year and species in the time series, we randomly sampled individuals from the size distribution data set until the sum of the planar area across colonies matched the coral cover reported in the time series data set (see methods in Carlot et al., 2021). We assumed that the planar area of the six species was approximately a circle, and we calculated individual planar areas from visually determined length and width (i.e., ((length + width)/4)^2^π). As a result, we defined a coral size distribution per taxa per year, and we scaled up the ratio between net photosynthesis and calcification rates for each hypothetical community over thirteen years. To strengthen our models, we repeated this 50 times, and we also ran the analysis without considering *Montipora*, *Napopora*, and *Astrea*, which did not change the results (Figure S3). All of the statistical analyses were run with the statistical software R version 4.0.3 (R Core Team, 2019).

## 3 RESULTS

**FIGURE 2:**
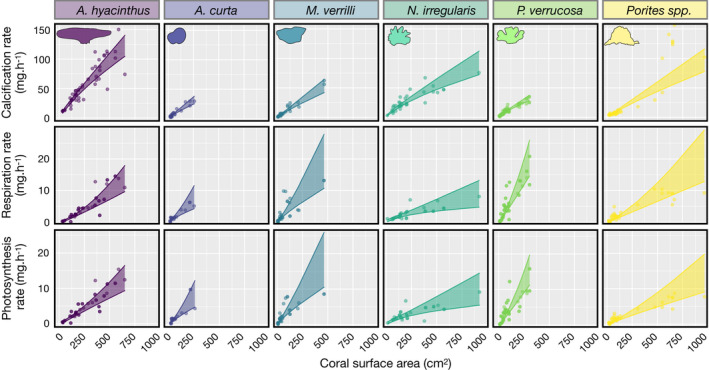
Scaling relationships between the three physiological processes (i.e., calcification, respiration, and photosynthesis rates) and live coral surface area for the six coral species (*Acropora hyacinthus*, *Astrea curta*, *Montipora verrilli*, *Napopora irregularis*, *Pocillopora* cf. *verrucosa*, and *Porites* spp.). Points represent the raw data, and regression lines represent posterior predictions from a Bayesian linear model (± 95% credible intervals). Coral silhouettes represent the mature coral morphologies of each species

**FIGURE 3:**
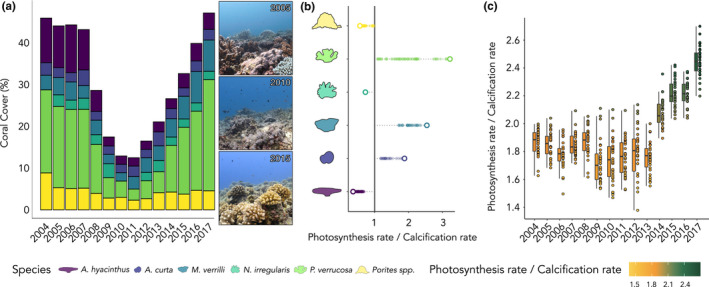
Hypothetical coral assemblages and their energy ratios (net photosynthesis rate/calcification rate). (a) Percentage of live coral cover of the 6 coral species from 2004 to 2017. Reefscapes are shown on the right for three years (i.e., 2005, 2010, and 2015). (b) The ratio between photosynthesis and calcification rates for the six coral species (*Acropora hyacinthus*, *Astrea curta*, *Montipora verrilli*, *Napopora irregularis*, *Pocillopora* cf. *verrucosa*, and *Porites* spp.). The solid vertical line represents the case where the photosynthesis rate is equal to the calcification rate. (c) The ratio between photosynthesis and calcification rates at the community level, from 2004 to 2017 within a theoretical 10 m^2^ transect

For all coral species, we observed an increase in individual calcification, respiration, and photosynthesis with increasing colony size (Figure 2). However, we identified both hypo‐allometric and isometric relationships, depending on the physiological process. Calcification showed hypo‐allometric relationships with colony size for each coral taxa, as evidenced by values of *β* that were lower than 1 (Tables 1 and 2). We found that smaller coral colonies calcify more efficiently, relative to their surface area. Although massive *Porites* spp., massive *A*. *curta*, and encrusting *M*. *verrilli* had higher *β* values than the other species, only 2% of the 5000 posterior draws had a slope equal or slightly greater than 1 (i.e., isometric trajectories), supporting that at the same area‐normalized rate, smaller coral colonies calcify faster. On the other hand, respiration and photosynthesis increased isometrically with colony size, as demonstrated by *β* values that did not differ from 1. We detected substantial among‐species variation in the *α* coefficients (i.e., intercepts) for all three physiological processes (Figure 2, Table 2). For example, *A*. *hyacinthus* showed the highest calcification rate per unit area, while *M*.* verrilli* exhibited the lowest calcification rate. Yet, this trend reversed for both respiration and photosynthesis, where *M*. *verrilli* and *A*. *hyacinthus* showed the highest and lowest rates, respectively. Depending on coral community composition around Mo'orea, these observations may have significant implications for large‐scale physiological processes (Figure 3a). Furthermore, we detected two main trends when examining species‐specific relationships between photosynthetic rates and calcification rates (Figure 3b). *Porites* spp., *N*. *irregularis*, and *A*. *hyacinthus* showed higher calcification rates than net photosynthetic rates, while *A*. *curta*, *M*. *verrilli*, and *P*. *verrucosa* showed the opposite pattern. Using these ratios to model population‐wide processes, we found that from 2004 to 2013, the average, community‐level ratio is fairly constant around 1.8, but after 2013, the average ratio significantly increased from 2.2 to 2.4 (Figure 3c).

**TABLE 2:** Estimates and 95% credible intervals for fitted parameters based on Bayesian linear models estimating calcification, respiration, and photosynthesis rates according to colony size for six coral species

Parameters	Calcification			Respiration			Photosynthesis		
	Mean	2.5%	97.5%	Mean	2.5%	97.5%	Mean	2.5%	97.5%
*A. hyacinthus*									
*α*	0.26	0.15	0.48	0.01	0.01	0.06	0.02	0.01	0.06
*β*	0.85	0.77	0.94	1.29	1.00	1.41	1.11	0.87	1.32
*A. curta*									
*α*	0.24	0.14	0.45	0.02	0.01	0.06	0.02	0.01	0.06
*β*	0.89	0.80	0.97	1.06	0.78	1.33	1.05	0.82	1.27
*M. verilli*									
*α*	0.24	0.14	0.45	0.02	0.02	0.07	0.02	0.01	0.06
*β*	0.93	0.83	1.00	1.00	0.71	1.26	0.98	0.74	1.19
*N. irregularis*									
*α*	0.24	0.14	0.45	0.01	0.01	0.06	0.02	0.01	0.05
*β*	0.82	0.75	0.91	0.76	0.47	1.02	0.80	0.56	1.01
*P. verrucosa*									
*α*	0.24	0.14	0.44	0.02	0.02	0.07	0.02	0.01	0.06
*β*	0.86	0.78	0.95	1.20	0.91	1.46	1.20	0.96	1.41
*Porites* spp.									
*α*	0.24	0.14	0.44	0.02	0.01	0.06	0.02	0.01	0.05
*β*	0.93	0.84	1.00	1.16	0.87	1.42	1.08	0.84	1.29

Notes

The coefficients *α* and *β* are calculated as metabolic rate = $\alpha S_{A}^{\beta }$, where S_A_ is the coral surface area (cm^2^) and the metabolic rate is expressed in (mg h^−1^). When *β* is lower than one, the metabolic rate scales hypo‐allometrically with the coral surface area, whereas when *β* equals 1, the metabolic rate scale isometrically with coral surface area.

## 4 DISCUSSION

### 4.1 Allometry vs. isometry scaling

We analyzed three physiological rates (i.e., calcification, respiration, and photosynthesis) for six prominent coral taxa to test whether the relationships between these rates and colony size are isometric or allometric. Similar to recent results (Carlot et al., 2021; Dornelas et al., 2017; Edmunds & Burgess, 2016; Madin et al., 2020), we found that calcification increases hypo‐allometrically per unit area with live coral surface area across all six taxa. However, this was not the case for photosynthesis and respiration, which scaled isometrically with live coral surface area. This contrasts with previous work, which suggests that respiration and photosynthesis in *Pocillopora* sp. scale hypo‐allometrically with colony size (Edmunds & Burgess, 2016). The prevalence of isometric relationships across the six species in our study suggests that isometric scaling of respiration and photosynthesis rates may be common across corals, at least at comparable, nonstressful environmental conditions (i.e., pH between 8.1 and 8.3 and temperature between 25.5°C and 30.2°C).

As opposed to the allometric scaling of calcification, the isometric scaling of photosynthesis emphasizes the importance of skeletal growth in early‐life stages. Small, recently settled colonies generally experience higher mortality rates (Penin et al., 2010; Ritson‐Williams et al., 2009; Wall & Stallings, 2018), and a rapid increase in colony size (through extensive calcification) may offer the best chance for survival (Doropoulos et al., 2012; Heino & Kaitala, 1999). Thus, while it is beneficial for small coral colonies to disproportionally invest in calcification, there are no immediate benefits from increased photosynthesis. In fact, high photosynthesis per unit surface area may hamper early‐life stage success through exposure to oxidative stress (Fitt et al., 2001; Hoogenboom & Anthony, 2006). Thus, photosynthetic energy may be allocated to others processes such as nutrient cycling (Falkowski et al., 1984), or it may be stored for reproduction at maturity (Leuzinger et al., 2003).

### 4.2 Physiological rates and energy allocation

Although we quantified ex situ calcification rates (using the alkalinity anomaly method), our results are consistent with those from other methods that determine coral growth, such as x‐rays (Lough, 2008), community metabolism (Langdon & Atkinson, 2005), and *in situ* measurements (Kuffner et al., 2013). *A*.* hyacinthus* had a consistently higher rate as compared to the other species. Our results support the high calcification rates documented for corals in the genus *Acropora*, which are classified as fast‐growing corals (Anderson et al., 2018; Harriott, 1999; Huston, 1985). Although *A*. *hyacinthus* had the highest calcification rate, its photosynthetic and respiratory rates were among the lowest in our experiments. This suggests that *A*. *hyacinthus* tends to allocate most of its energy to growth, at least in the absence of spawning activity, during which large amounts of energy may be dedicated to gamete development (Razak et al., 2020). Conversely, *M*. *verrilli* and *P*. *verrucosa* had the highest photosynthetic rates (Figure 2, Figure S2) but markedly lower calcification rates than *A*. *hyacinthus*, which highlights differences in the life‐history strategies of the various species (e.g., reproduction strategies). For pocilloporids, brooding sperm and egg bundles may require this energetic investment and subsequently enhance the chances of *Pocillopora* offspring to survive (Hirose et al., 2001). Indeed, the high photosynthetic rate of *P*. *verrucosa* may explain the success of this species in Mo'orea, a reef system increasingly dominated by pocilloporids (Hédouin et al., 2020). Although *M*. *verrilli* is a broadcast spawner, it is the second most abundant coral genus in Mo'orea (Bosserelle et al., 2014), suggesting that higher photosynthesis rates are directly related to species’ perennity under current environmental conditions.

Notably, *M*. *verrilli* and *P*. *verrucosa* are also known for their lower *Symbiodinium* density (Edmunds et al., 2014; Putnam & Edmunds, 2011, Coral Trait Database), which may support their high photosynthetic rates. The distinct photosynthetic rates among coral taxa might arise from the different physiological and ecological attributes of associated symbiotic communities (Baird et al., 2009; Putnam et al., 2012; Rouzé et al., 2019). Thus, the present community composition around Mo'orea suggests that the physiological profile of *A*. *hyacinthus* and its variable symbionts are at a disadvantage under current conditions, as the genus has become rare as compared to *P*. *verrucosa* or *M*. *verrilli* (Babcock et al., 2003).

### 4.3 Limitations and scaling recommendations

Our study focused on current *in situ* conditions (i.e., low cloud cover, low sedimentation, temperatures lower than 30°C, pH *ca*. 8.2); therefore, additional work is required to strengthen the robustness of our findings and affirm our predictions for future coral communities under global change (e.g., ocean warming, increases in storm intensity). Indeed, light intensity and water flow highly impact physiological rates, and they may significantly affect calcification rates (Cresswell et al., 2020; Edmunds & Burgess, 2017). Moreover, the measurements in the present study were carried out from September to December, so seasonality was not considered. Finally, our findings are derived from a distinct size spectrum of corals. Specifically, our work focused on relatively small coral colonies that are dominant after severe disturbances, such as cyclones (Carlot et al., 2021); thus, our findings may have biases through the omission of larger, more mature colonies.

Understanding the nature of the investigated scaling relationships opens opportunities to estimate ecosystem‐wide processes that are critical for coral reef functioning. In the case of photosynthesis and respiration, isometric scaling permits relatively simple extrapolations of colony‐level processes to entire communities. Specifically, if species identities and the relative combined surface areas of colonies are known, we may be able to compute estimates of community‐wide respiration and photosynthesis. In contrast, due to the size dependency of calcification, community‐level calcification estimations would require information on the size distributions of individual colonies, which are seldom recorded in standard monitoring (e.g., photo‐quadrats, point counts; Edmunds & Riegl, 2020). Given that calcification has direct implications for reef accretion (Perry et al., 2018) and wave energy attenuation (Harris et al., 2018), the absence of colony size from most major coral reef monitoring programs may preclude us from inferring community‐level processes with adequate accuracy.

Moreover, the observed ratio between net photosynthesis and calcification rates supports the idea that coral demography may be an important determinant of community functioning. However, our results are only based on coral‐cover variation. The size distributions of coral colonies were kept constant among coral species (Kayal et al., 2018), and, therefore, they may display different trajectories when colony size variation is accounted for, especially for processes that follow allometric scaling (Carlot et al., 2021). In order to scale from individual to community‐level physiological rates, we recommend prioritizing photogrammetric monitoring, which allows the definition of both coral cover and coral colony size (Kornder et al., 2021).

### 4.4 Conclusion

Overall, our results expand our understanding of coral physiology and species‐specific traits that can confer ecological advantages under changing environmental conditions. Further, our findings strengthen our capacity to predict community‐wide photosynthesis rates and respiration based on traditionally collected coral cover survey data. Our results suggest that the lack of demographic data (i.e., colony size) across the literature and many monitoring databases prevents us from defining community‐wide estimates of calcification. Therefore, including colony size would greatly enhance long‐term monitoring efforts.

## CONFLICT OF INTEREST

None declared.

## AUTHOR CONTRIBUTION

**Jeremy Carlot:** Data curation (equal); Formal analysis (equal); Investigation (equal); Validation (equal); Visualization (equal); Writing – original draft (equal); Writing – review & editing (equal). **Héloïse Rouzé:** Supervision (equal); Validation; Writing – review & editing. **Diego R. Barneche:** Conceptualization (equal); Formal analysis; Validation (equal); Writing – review & editing (equal). **Alexandre Mercière:** Data curation; Investigation; Methodology (equal); Writing – review & editing. **Benoit Espiau:** Data curation; Investigation; Methodology (equal); Writing – review & editing. **Ulisse Cardini:** Data curation; Investigation; Methodology; Writing – review & editing. **Simon J. Brandl:** Resources (lead); Writing – review & editing (equal). **Jordan M. Casey:** Writing – review & editing. **Gonzalo Pérez‐Rosales:** Writing – review & editing. **Mehdi Adjeroud:** Writing – review & editing (equal). **Laetitia Hédouin:** Funding acquisition (equal); Writing – review & editing (equal). **Valeriano Parravicini:** Conceptualization (equal); Funding acquisition (equal); Resources (equal); Supervision (equal); Validation; Writing – review & editing (equal).

### OPEN RESEARCH BADGES

This article has been awarded Open Materials, Open Data Badges. All materials and data are publicly accessible via the Open Science Framework at https://github.com/JayCrlt/Coral_Physiology.

## Supporting information

Figure S1‐S3

## Data Availability

Code and data are available at https://github.com/JayCrlt/Coral_Physiology.
