# Supplementary material for: Scaling up calcification, respiration, and photosynthesis rates of six prominent coral taxa
Source: Ecol Evol. 2022 Mar 18;12(3):e8613. doi: 10.1002/ece3.8613 (PMC8933251; doi:10.1002/ece3.8613)
Supplement: Supplementary file 1 — Figure S1‐S3 [file ECE3-12-e8613-s001.pdf]

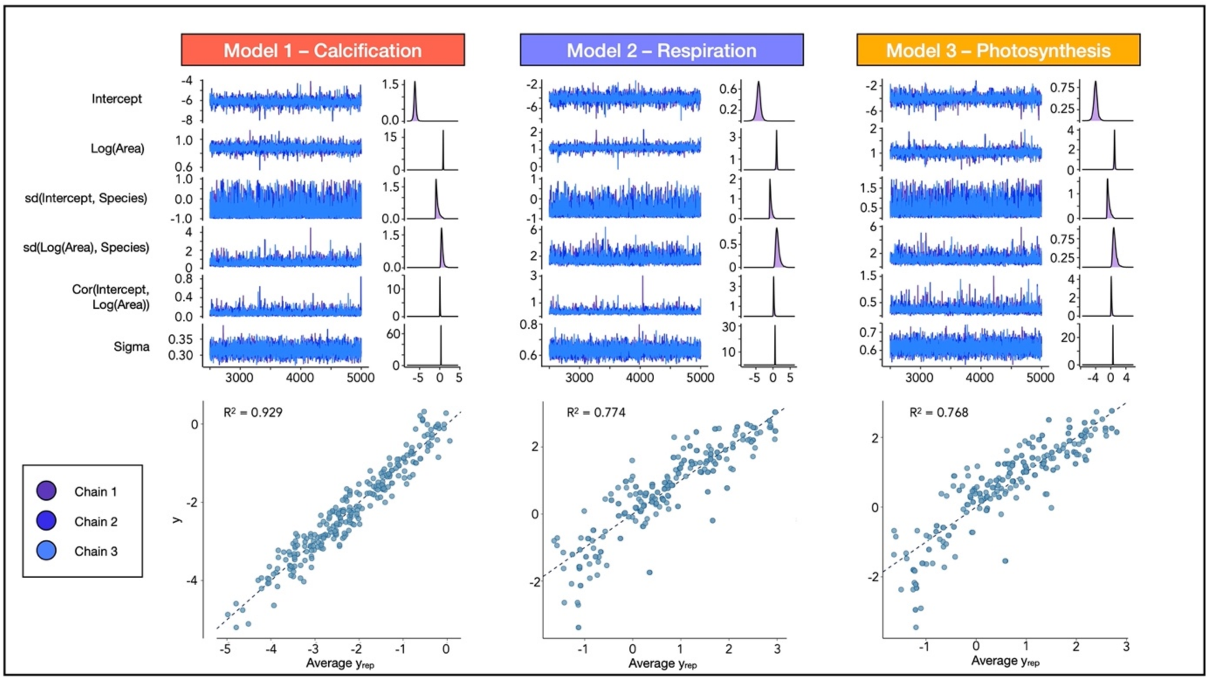

Figure S1 | Trace plots depicting the Monte Carlo chains from the three Bayesian models. Each model (*i.e.,* calcification, respiration and photosynthesis) was run with three chains of 5,000 iterations, with the first 2,500 steps discarded. The scatterplots represent posterior predictive checks and include the respective R^2 values estimated from the model.

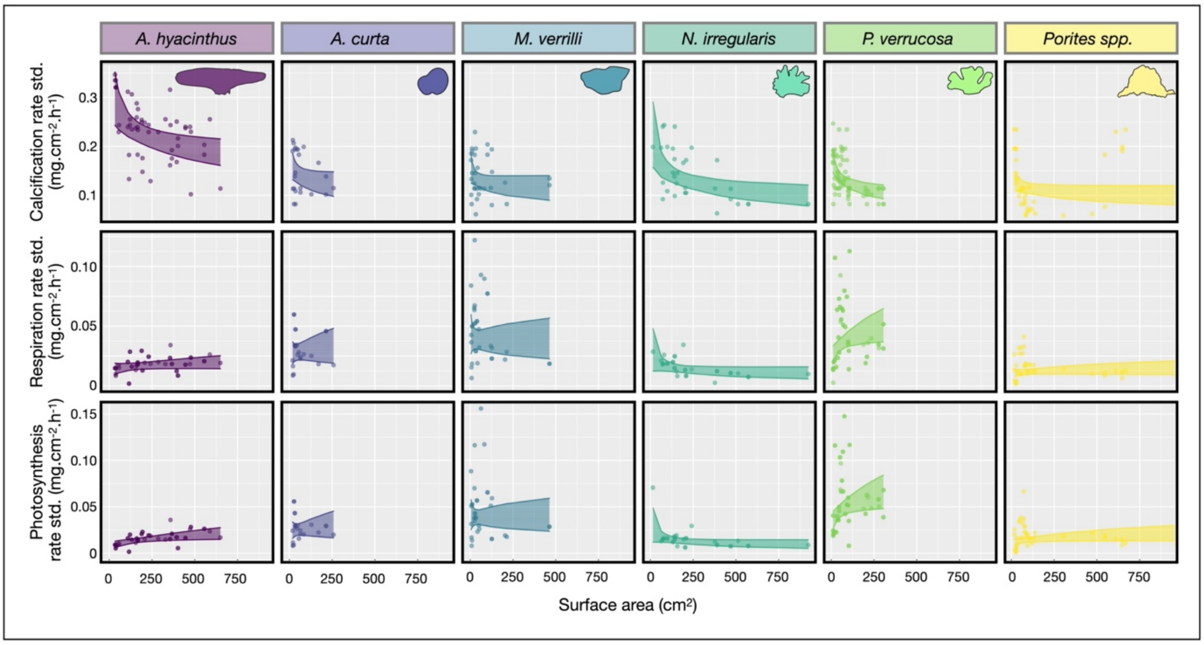

Figure S2 | The relationship between the surface area-specific physiological processes (calcification, respiration and photosynthesis rates, respectively, from top to bottom) and live coral surface area for the six coral species (*Acropora hyacinthus, Astrea curta, Montipora verrilli, Napopora irregularis, Pocillopora* cf. *verrucosa* and *Porites spp.*) with a ± 95% Bayesian credible interval. Points represent the raw data, and the regression lines represent posterior predictions from the Bayesian linear model (± 95% credible intervals). Coral silhouettes represent mature coral morphologies of each species.

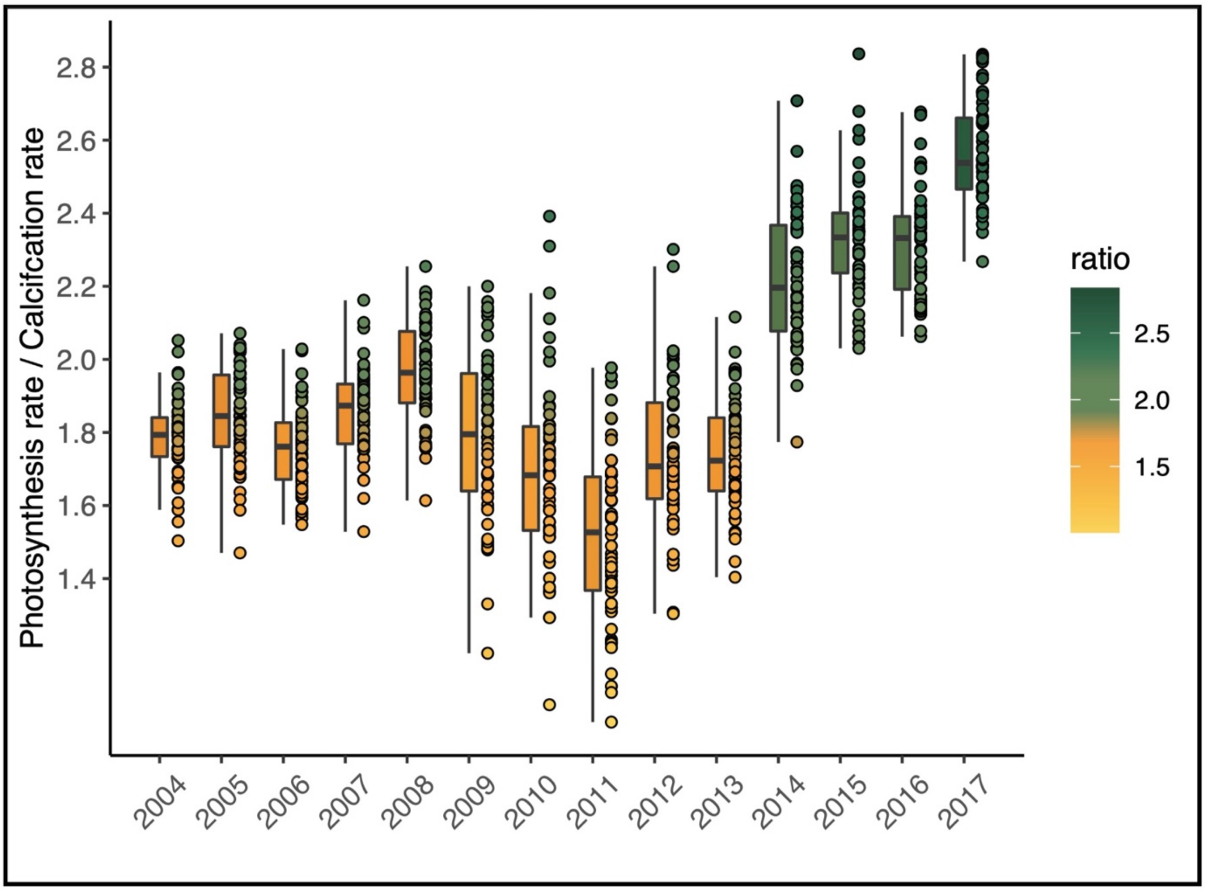

Figure S3 | The ratio between photosynthesis and calcification rates at the community level, from 2004 to 2017 within a theoretical 10m^2 transect, considering only *Acropora*, *Pocillopora* and *Porites*.
